# An exploratory analysis of head-tilting in dogs

**DOI:** 10.1007/s10071-021-01571-8

**Published:** 2021-10-26

**Authors:** Andrea Sommese, Ádám Miklósi, Ákos Pogány, Andrea Temesi, Shany Dror, Claudia Fugazza

**Affiliations:** 1 Department of Ethology, Eötvös Loránd University, Budapest, Hungary; 2 MTA-ELTE Comparative Ethology Research Group, Budapest, Hungary

**Keywords:** Dogs, Head-tilt, Human–dog communication, Laterality

## Abstract

Little is known about head-tilts in dogs. Based on previous investigations on the head turning and the lateralised brain pattern of human speech processing in dogs, we hypothesised that head-tilts may be related to increased attention and could be explained by lateralised mental functions. We observed 40 dogs during object-label knowledge tests and analysed head-tilts occurring while listening to humans requesting verbally to fetch a familiar toy. Our results indicate that only dogs that had learned the name of the objects tilted their heads frequently. Besides, the side of the tilt was stable across several months and tests. Thus, we suggest a relationship between head-tilting and processing relevant, meaningful stimuli.

**Supplementary Information:**

The online version contains supplementary material available at 10.1007/s10071-021-01571-8.

## Introduction

Several vertebrates (e.g. fish, reptiles, birds, and mammals) process sensory information asymmetrically (Rogers et al. 2013). Earlier observations showed different manifestations of lateralisation in dogs, such as asymmetry in tail wagging (Siniscalchi et al. 2017), nostril use (Siniscalchi et al. 2016), and pawedness (Ocklenburg et al. 2019).

Dogs focus their visual and/or acoustic attention reflecting lateralisation in brain functioning (Reinholz-Trojan et al. 2012; Siniscalchi et al. 2008, 2010). Ratcliffe and Reby (2014) showed that dogs consistently turn their head slightly to the left during the presentation of a familiar spoken command, while a right bias was observed in response to manipulated, meaningless stimuli. Andics et al. (2016, 2014a, b, 2017), together with Gabor et al. (2020), confirmed through neuroimaging the existence of a brain specialisation in dogs for processing speech with a right-hemisphere bias for praise words.

**Fig. 1:**
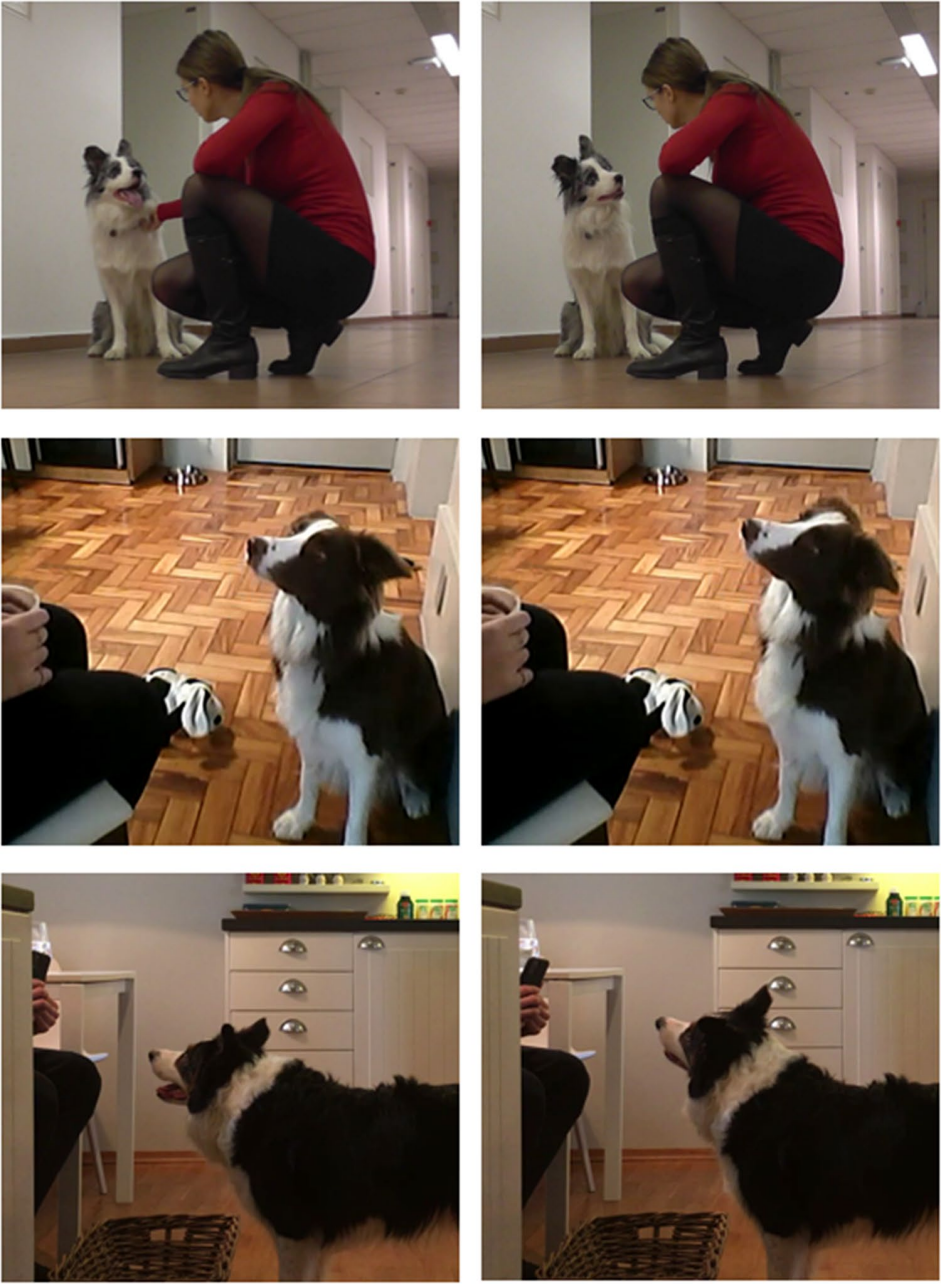
From top to bottom: Max, Gaia, and Whisky just before and while performing a head-tilt (left and right photos, respectively) during Experiments 1 and 2

Another asymmetrical head movement in dogs is the head-tilt. Tilting is a lateral, horizontal movement of the head out of the vertical plane (Fig. 1). To the best of our knowledge, no study has described head-tilting in dogs. We investigated the occurrence and direction of the head-tilts dogs perform in response to human verbal vocalisations, specifically, when the dogs were asked to fetch a toy using its trained label.

Only a few dogs can learn the name of objects (toys) even after a few exposures, while most (typical) dogs do not (Fugazza et al. 2021a, b). We define the dogs that rapidly learn object labels as gifted word learner (*GWL*) dogs (Dror et al. 2021). We expected that, if head-tilting is related to processing meaningful or relevant auditory stimuli, dogs that learn object labels would tilt their heads more frequently upon hearing the toy’s name than typical dogs. Dogs displaying a consistent preference for one side over time would suggest asymmetric processing of the verbal stimuli (Wells et al. 2018). Alternatively, the lack of a population-level bias would support that head-tilting represents a habitual, idiosyncratic behaviour related to attention.

## Materials and methods

Ethical permission for conducting this study was obtained from The Institutional Committee of Eötvös Loránd University (N. PE/EA/691-5/2019). All owners gave informed consent to participate in the study.

### Subjects

We recruited 40 dogs: 33 family dogs motivated for toys (typical dogs) and 7 dogs that were consistently successful in learning object names (*GWL* dogs); Data collection on the head-tilts was carried out as the subjects were involved in another study in which they were trained to learn object names (Fugazza et al. 2021b). The *GWL* dogs also participated in another study on learning and memory consolidation of object names (Dror et al. 2021).

### Procedure

#### Experiment 1: monthly tests, 2 toys

We observed head-tilts in all dogs (*N* = 40) tested on object-label acquisition after 1, 2, and 3 months from the beginning of a 3-month-long training program aimed at teaching them the name of 2 novel toys (Fugazza et al. 2021b). Each dog had a consistent pair of toys to learn during the training period. During the test, the owner asked the dog to fetch one of the toys (randomly determined) by pronouncing its name (e.g. “bring rope!”). The dogs were standing or sitting in front of the owner while the toys were in an adjacent room. Upon hearing the owner’s request, the dogs entered the room, chose a toy, and brought it back to the owner. Each of the monthly tests (3 in total) consisted of 12 trials per dog, using the same pair of toys throughout each test.

#### Experiment 2: monthly tests, multiple toys

In experiment 2, only dogs that were able to learn the names of the two toys trained in Experiment 1 above the chance-level were further tested (see also Fugazza et al. 2021b). For this reason, only the 6 *GWL* and none of the typical dogs were included in this test (one *GWL* dog could not be included as she passed away). The procedure was similar to that described previously but it included all new toys that the owners had introduced to their dogs. The number of toys laid on the floor across the tests varied for each dog, based on how many new toys the dogs learned (1st month, 2–11; 2nd month, 3–12; and 3^rd^ month, 2–13 toys). Each toy was randomly requested twice, and the number of total trials varied from dog to dog (Gaia: 28 trials; Max: 15; Nalani: 37; Rico: 16; Squall: 20; and Whisky: 59 trials).

#### Experiment 3: genius dog challenge

The 6 *GWL* dogs also participated in Experiment 3. The starting date for this experiment was the same for all the *GWL* dogs, 2–10 months after Experiment 2, based on when each dog finished the testing program for that study. They had 7 days to learn the names of 6 new toys in the first phase of the experiment, and 12 additional toys in the second. In both phases, on the seventh day, they were tested for their learning outcome (Dror et al. 2021). The testing procedure and setup were identical to those described above (i.e. all toys scattered on the floor in one room, the owner and the dog in another, with the owner requesting the toys verbally one by one in a randomised order). The two phases consisted of 15 and 27 trials per dog, respectively.

## Behavioural data collection

For every trial, the display (or absence) of head-tilt was noted from when the owner started to speak to the dog until when the dog left to go fetch a toy. We reported the direction of the movement and the position of the owner. For both, we considered the dog’s midline as a reference for direction and identified 3 possible relative positions of their owners (i.e. left, in front of, right). Head-tilting was defined as follows: the dog cocks the head on either side (Fig. 1). 20% of the videos were coded by an independent coder for inter-rater agreement.

## Data analysis

See Supplementary Material.

## Results

**Fig. 2:**
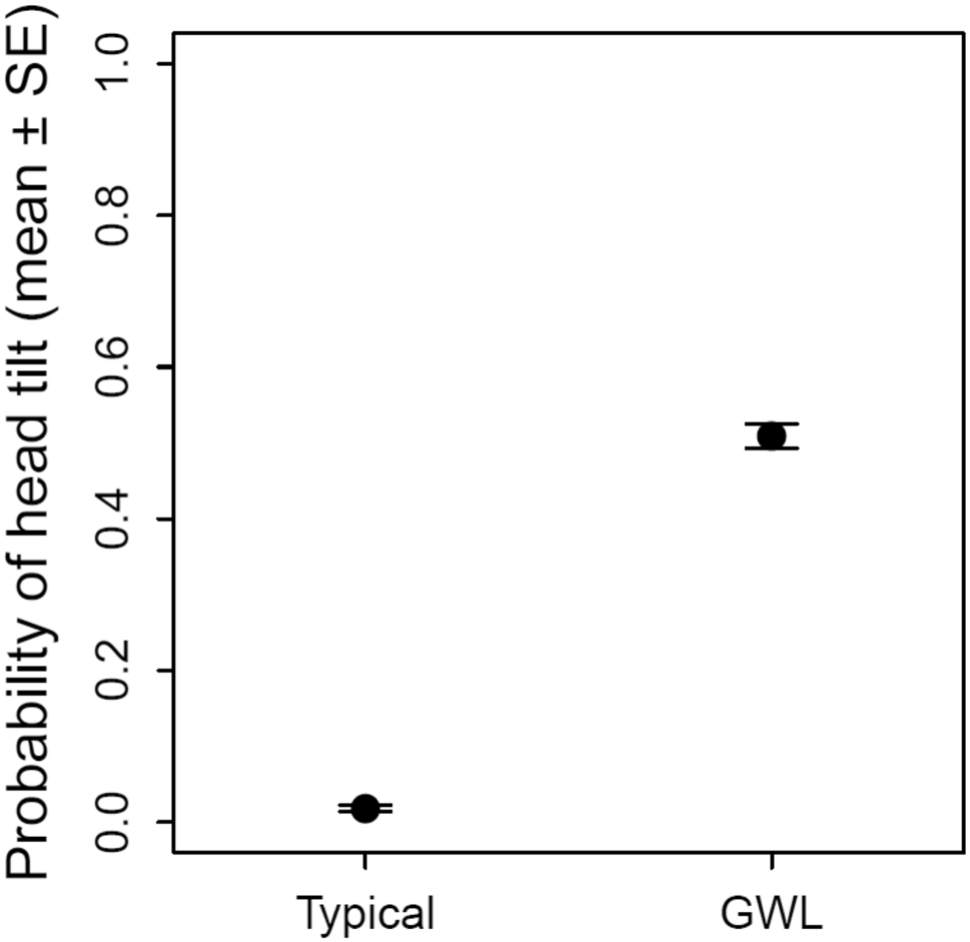
Average probability (± SE) of head-tilting (Experiment 1, *N* = 33 typical and 7 *GWL* dogs)

Inter-rater agreement was excellent (Cronbach’s alpha > 0.9). In Experiment 1, the *GWL* dogs tilted their heads significantly more frequently than *typical* dogs (43% vs 2% of trials; binomial GLMM, LRT of experimental group: *χ*^2^ = 23.847, d*f* = 1, *p* < 0.001; Fig. 2). Only one of the *GWL* dogs never tilted his head (see Supplementary Material).

**Fig. 3:**
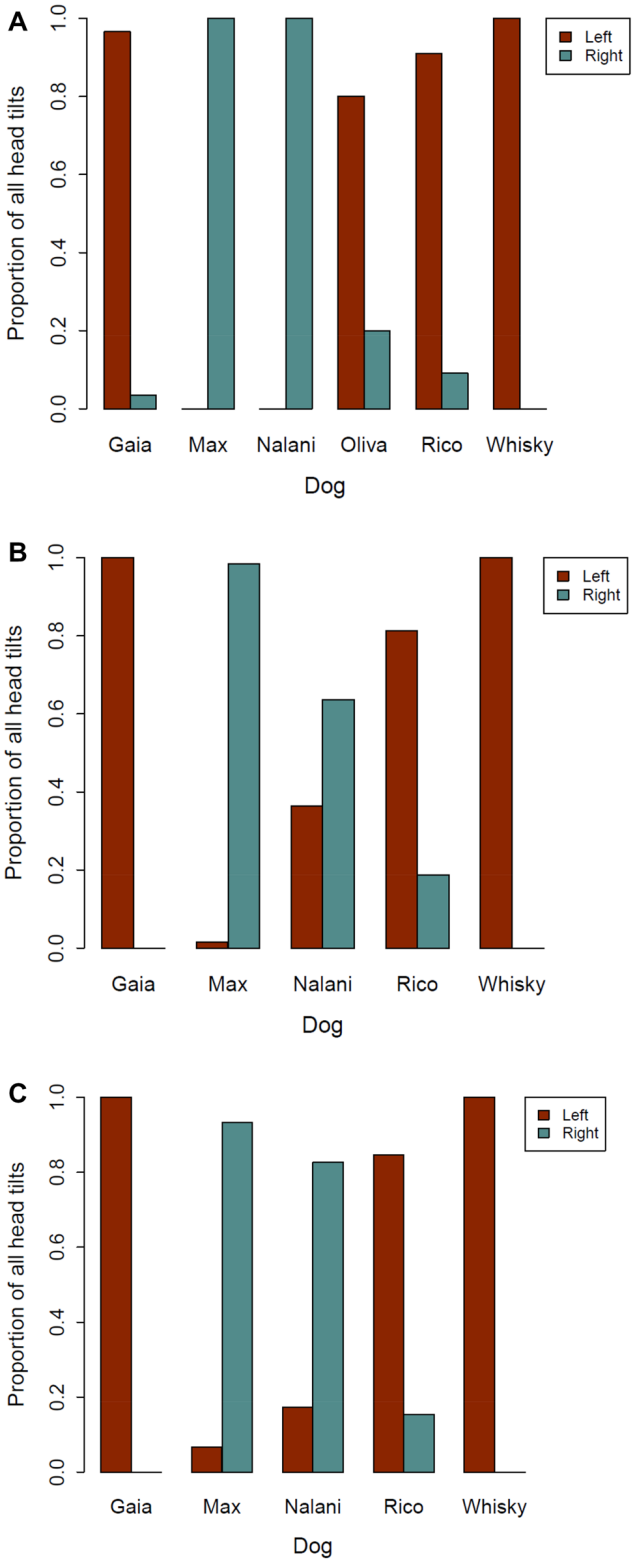
Proportion of head-tilts to each direction by the *GWL* dogs in Experiment 1 (**A**), Experiment 2 (**B**), and Experiment 3 (**C**). Oliva was tested only in Experiment 1

In Experiment 1, the direction of head-tilt was significantly repeatable across the monthly tests (*r* = 0.738 ± 0.27, *p* < 0.001). Consistent results were also found in both Experiments 2 (*r* = 0.841 ± 0.28, *p* < 0.001), and 3 (*r* = 0.801 ± 0.24, *p* < 0.001) (Fig. 3).

In all trials, head-tilting occurred when the dogs were oriented towards the owner. The owner’s relative position to that of the dog did not correlate with the direction of head-tilt (Pearson’s correlations in Experiments 1–3: all *p* > 0.494).

## Discussion

Upon hearing the owners’ request for a familiar toy, the *GWL* dogs tilted the head significantly more than typical dogs (“Experiment 1”). Importantly, typical dogs were equally familiarised with the spoken object names, as all dogs had been exposed to 3 months of training with them, all owners applied the same training protocol and received the same instructions during weekly training sessions with a dog trainer (Fugazza et al. 2021b). Thus, in the context of object verbal labels, the familiarity of the stimulus alone was not enough to elicit head-tilts. Therefore, we suggest that the difference in the dogs’ behaviour might be related to hearing meaningful words (for the *GWL* dogs) and could be a sign of increased attention. Possibly, head-tilts could also be related to making a cross-modal match in the dogs’ memory (e.g. name to a visual image) upon hearing the toy’s name.

The position of the owner did not influence the side of the head-tilt. Hence, the location of the sound source can be excluded as a confounding factor.

The direction of the head-tilts was individually consistent across the different studies, revealing that the direction of the tilt should be considered as a stable individual trait. This observation is in line with previous findings of paw preference in dogs. For instance, Wells et al. (2018) reported task-specific paw use in dogs where the subjects displayed the same pawedness after 6 months when tested again under the same conditions.

There is evidence for lateralisation in processing human vocalisations in the dog brain (Andics et al. 2014a; b) but the small number of *GWL* dogs in this study hinders investigating a population-level side bias. Future studies with a larger sample size may combine behavioural and neural approaches to reveal the relationship between the direction of head-tilting and neural processing of human vocalisations.

All the 6 *GWL* dogs performing frequent tilts in this study were Border collies. However, the majority of typical dogs not displaying such behaviour also belonged to this breed (*N* = 18). Hence, it is important to refrain from relating frequent head-tilts with Border collies. Most *GWL* dogs reported in the literature belong to this breed but the vast majority of Border collies do not appear to have the capacity to learn object names (Fugazza et al. 2021b). It is also important to note that a few dogs of other breeds have shown this skill (Fugazza et al. 2021a; Griebel and Oller 2012; Ramos and Ades 2012). Since the frequency of head-tilts in *GWL* dogs of other breeds has not been studied, further work is needed to address the generalizability of the present results to other breeds.

## Supplementary Information

Below is the link to the electronic supplementary material.

Supplementary file1 (DOCX 45 KB)
