# Supplementary material for: An exploratory analysis of head-tilting in dogs
Source: Anim Cogn. 2021 Oct 26;25(3):701–5. doi: 10.1007/s10071-021-01571-8 (PMC9107419; doi:10.1007/s10071-021-01571-8)
Supplement: Supplementary file 1 — Supplementary file1 (DOCX 45 KB) [file 10071_2021_1571_MOESM1_ESM.docx]

# Supplementary material: An Exploratory Analysis of Head-Tilting in Dogs

Andrea Sommese, Ádám Miklósi, Ákos Pogány, Andrea Temesi, Shany Dror, Claudia Fugazza

Methods

*Subjects*

**Typical dogs**

Of the 33 dogs we recruited (59% males), 17 dogs belonged to various breeds (age: 43 ± 35.79 weeks) and 16 were Border collies (age: 28 ± 22.43 weeks). The owners of the dogs volunteered for the study. All of these dogs, apart from three adult Border collies, were recruited in Budapest (Hungary), through the Family Dog Project database. The other three Border collies were recruited in Brazil. None of the 33 dogs have shown any evidence of learning the name of the toys during 3 months of intensive training and testing (Fugazza et al., 2021b).

**Gifted word-learner dogs (GWL dogs)**

N = 7 dogs that had shown the ability to learn object labels (all Border collies: 4 females, and 3 males; age: 44.3 ± 29.54 weeks) (Fugazza et al., 2021b). Six of these dogs had previous knowledge of 15-59 object names. Oliva did not have previous vocabulary knowledge but during the first two months of the training and testing program, she acquired knowledge of 21 object names (Fugazza et al., 2021b). Unfortunately, Oliva could only be tested once (in Experiment 1) because she passed away afterwards.

**Data analysis**

Statistical analyses were carried out using the R statistical environment (version 4.0.5). Cronbach’s alpha was used to assess inter-rater agreement between the two independent coders (DeVillis, 1991). The frequency and direction of head-tilts (response variables) were analysed in separate generalized linear mixed models (GLMM; ‘glmer’ function of R-package ‘lme4’; (Bates, Mächler, Bolker, & Walker, 2015). Test and Trial were included in initial models and were kept only if they had a significant effect. We provided statistics for likelihood ratio tests (LRT) between models including and excluding the given term. We also calculated repeatability estimates using bootstrapping (‘rpt’ function of R-package ‘rptR’; (Stoffel, Nakagawa, & Schielzeth, 2017) to investigate within-individual consistency in frequency and direction of head-tilts. We checked whether the direction of the tilt was correlated to the owner’s position, the latter calculated as a position score (0 – left, 0.5 – in front of, 1 – right). Finally, we calculated the consistency of the direction of tilts across conditions.

Results

Table 1: Percentage of trials in which the dogs displayed head-tilts (out of 12 trials) and breed of the dogs showing this behaviour in Experiment 1. Only those tests are reported in which the target behaviour was observed (tests of 12 typical dogs out of 40, and 6 GWL dogs out of 7).

| **Name** | **Breed** | **Type** | **HT** | **Test** |
| --- | --- | --- | --- | --- |
| Athena | Border collie | TYPICAL | 8% | 2 months |
| Benhur | Kelpie | TYPICAL | 16% | 3 months |
| Enzo | Whippet | TYPICAL | 8% | 3 months |
| Kesu | Border terrier | TYPICAL | 8% | 2 months |
| Kiki | Border collie | TYPICAL | 8% | 2 months |
| Liza | Vizsla | TYPICAL | 8% | 2 months |
| Lizy | Border collie | TYPICAL | 8% | 2 months |
| Lizy | Border collie | TYPICAL | 8% | 3 months |
| Nola | Border collie | TYPICAL | 8% | 3 months |
| Popi | Border collie | TYPICAL | 8% | 3 months |
| Roza | Border collie | TYPICAL | 8% | 3 months |
| Scotch | Australian shepherd | TYPICAL | 8% | 2 months |
| Zeno | Border collie | TYPICAL | 16% | 2 months |
| Zeno | Border collie | TYPICAL | 16% | 3 months |
| Gaia | Border collie | GWL | 92% | 1 month |
| Gaia | Border collie | GWL | 83% | 2 months |
| Gaia | Border collie | GWL | 67% | 3 months |
| Max | Border collie | GWL | 67% | 1 month |
| Max | Border collie | GWL | 42% | 2 months |
| Max | Border collie | GWL | 33% | 3 months |
| Nalani | Border collie | GWL | 8% | 1 month |
| Nalani | Border collie | GWL | 16% | 2 months |
| Oliva | Border collie | GWL | 42% | 1 month |
| Rico | Border collie | GWL | 42% | 1 month |
| Rico | Border collie | GWL | 92% | 2 months |
| Rico | Border collie | GWL | 16% | 3 months |
| Whisky | Border collie | GWL | 83% | 1 month |
| Whisky | Border collie | GWL | 50% | 2 months |
| Whisky | Border collie | GWL | 92% | 3 months |

Table 2: Percentage of trials in which the dogs displayed head-tilts in Experiment 2. The total number of trials in this experiment varies according to the number of toys each subject was able to fetch reliably at the time. Only the tests in which the target behaviour was observed are reported.

| Gaia | Border collie | GWL | 68% | 1 month |
| --- | --- | --- | --- | --- |
| Gaia | Border collie | GWL | 91% | 2 months |
| Gaia | Border collie | GWL | 96% | 3 months |
| Max | Border collie | GWL | 71% | 1 month |
| Max | Border collie | GWL | 54% | 2 months |
| Max | Border collie | GWL | 76% | 3 months |
| Nalani | Border collie | GWL | 42% | 1 month |
| Nalani | Border collie | GWL | 3% | 2 months |
| Rico | Border collie | GWL | 19% | 1 month |
| Rico | Border collie | GWL | 69% | 2 months |
| Rico | Border collie | GWL | 10% | 3 months |
| Whisky | Border collie | GWL | 96% | 2 months |
| Whisky | Border collie | GWL | 97% | 3 months |

Table 3: Percentage of trials in which the dogs displayed head-tilts in Experiment 3. The number of the trials varies between 15 and 27, following the two stages of the Genius Dog Challenge [(http://www.geniusdogchallenge.com/](http://www.geniusdogchallenge.com), Dror. 2021). Only the tests in which the target behaviour was observed are reported.

| Gaia | Border collie | GWL | 73% | 1 GDC |
| --- | --- | --- | --- | --- |
| Gaia | Border collie | GWL | 70% | 2 GDC |
| Max | Border collie | GWL | 73% | 1 GDC |
| Max | Border collie | GWL | 70% | 2 GDC |
| Nalani | Border collie | GWL | 33% | 1 GDC |
| Nalani | Border collie | GWL | 67% | 2 GDC |
| Rico | Border collie | GWL | 60% | 1 GDC |
| Rico | Border collie | GWL | 15% | 2 GDC |
| Whisky | Border collie | GWL | 87% | 1 GDC |
| Whisky | Border collie | GWL | 78% | 2 GDC |
